# Supplementary material for: Non-Targeted Metabolome Analysis with Low-Dose Selenate-Treated Arabidopsis
Source: Plants (Basel). 2025 Jan 22;14(3):322. doi: 10.3390/plants14030322 (PMC11820405; doi:10.3390/plants14030322)
Supplement: Supplementary file 1 [file plants-14-00322-s001.zip › plants-3407532-Supplemental Files_Li-highlight.pdf]

Supplemental Table S3 Primers used for quantitative RT-PCR

Gene name	AGI	Forward primer 5'–3'	
BGLU28	AT2G44460	BGLU28_qF	TTGCCACTGAACTAGATTGGCA
		BGLU28_qR	GGATCGGCTTGTGGAATATGAG
APR3	AT4G21990	APR3_qF	GGAATCCATTGTTGCTTCTGAGGT
		APR3_qR	CAGAGCAACATCTTCAGCTCCACT
SULTR1:1	AT4G08620	SULTR1;1_qF	GCCATCACAATCGCTCTCCAA
		SULTR1;1_qR	TTGCCAATTCCACCCATGC
SULTR1:2	AT1G78000	SULTR1;2_qF	GGATCCAGAGATGGCTACATGA
		SULTR1;2_qR	TCGATGTCCGTAACAGGTGAC

Supplemental Figure S1

A

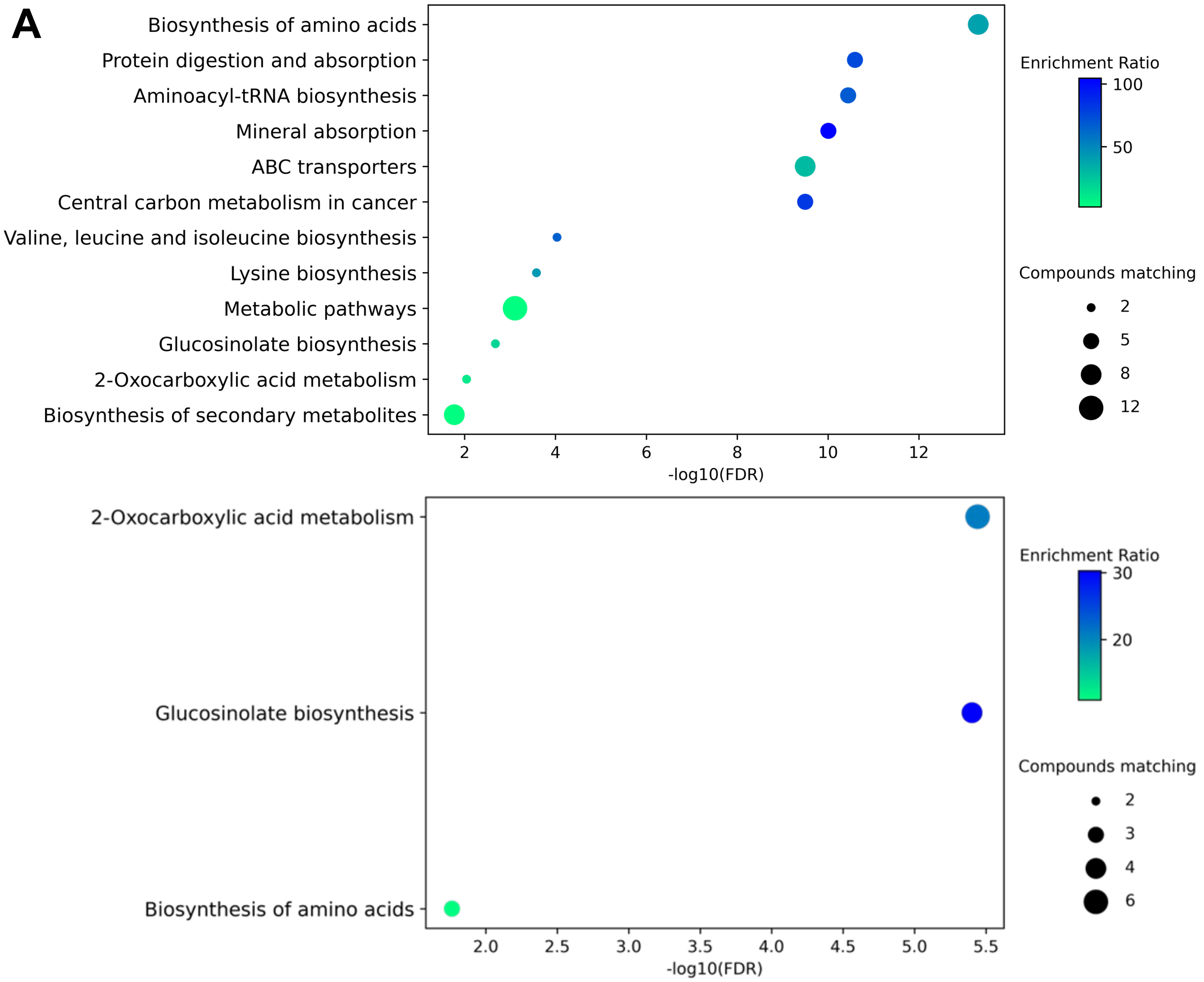

B

Enriched metabolic pathway (upregulated)_positive	Factor	-log10 (FDR)	-log10 (p)
Biosynthesis of amino acids	3.95	13.31	15.11
Protein digestion and absorption	7.55	10.59	12.09
Aminoacyl-tRNA biosynthesis	6.81	10.44	11.76
Mineral absorption	10.47	10.01	11.20
ABC transporters	2.86	9.50	10.60
Central carbon metabolism in cancer	8.21	9.50	10.52
Valine, leucine and isoleucine biosynthesis	5.49	4.03	4.99
Lysine biosynthesis	3.61	3.58	4.43
Metabolic pathways	2.53	3.11	3.91
Glucosinolate biosynthesis	1.97	2.68	3.40
2-Oxocarboxylic acid metabolism	1.13	2.04	2.70
Biosynthesis of secondary metabolites - part 2	2.57	1.77	2.27
Microbial metabolism in diverse environments	2.53	1.27	1.46

Enriched metabolic pathway (upregulated)_negative	Factor	-log10 (FDR)	-log10 (p)
2-Oxocarboxylic acid metabolism	25.84	3.94	5.12
Glucosinolate biosynthesis	34.26	3.30	4.18
Biosynthesis of secondary metabolites	2.03	0.91	1.27
Metabolic pathways	0.90	0.14	0.14

C

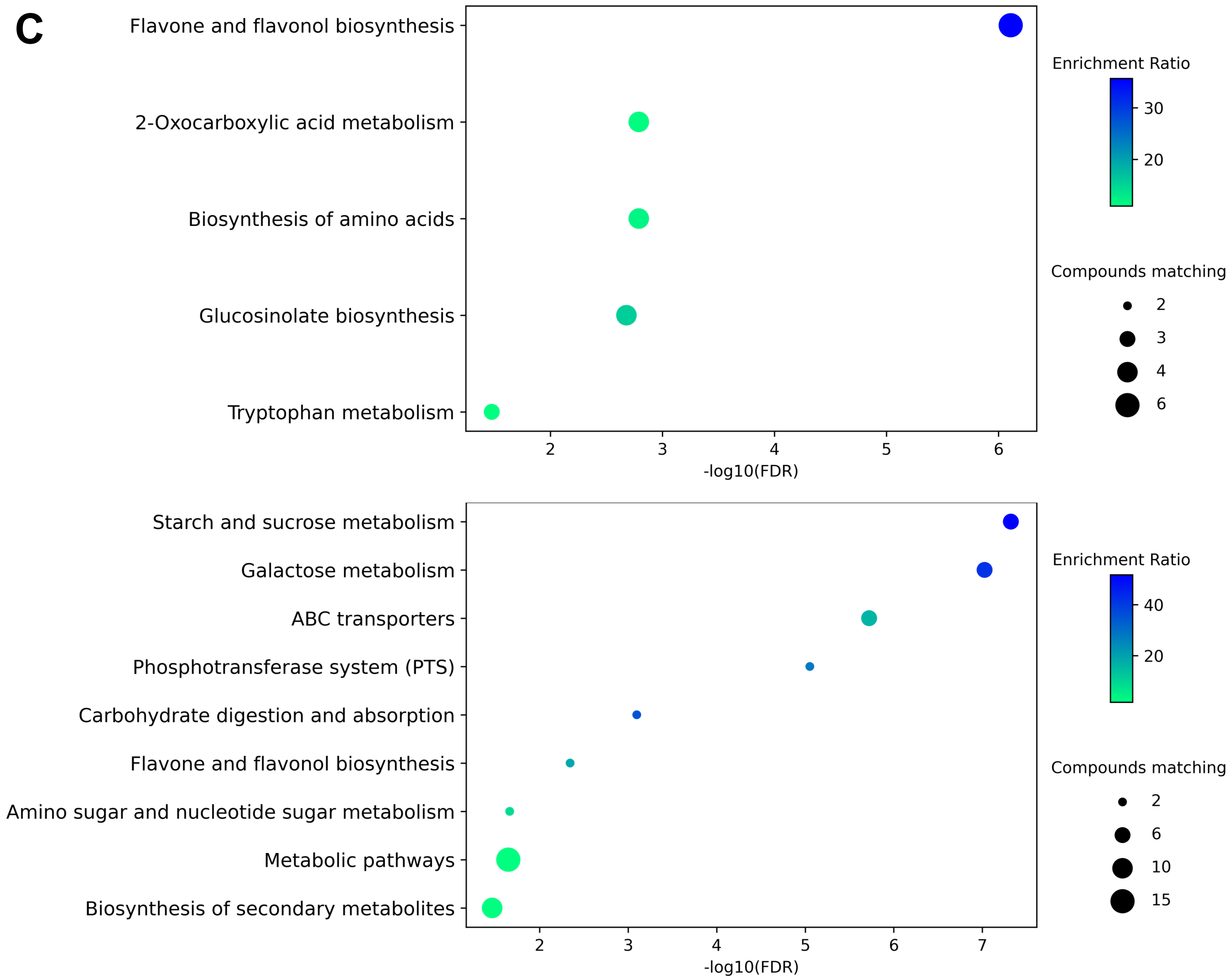

D

Enriched metabolic pathway (downregulated)_positive	Factor	-log10 (FDR)	-log10 (p)
Flavone and flavonol biosynthesis	36.57	6.11	8.03
2-Oxocarboxylic acid metabolism	11.34	2.79	4.24
Biosynthesis of amino acids	11.80	2.79	4.33
Glucosinolate biosynthesis	15.73	2.68	4.00
Tryptophan metabolism	9.10	1.48	2.62
Biosynthesis of secondary metabolites	15.79	1.00	1.29
Biosynthesis of cofactors	2.80	0.87	1.05
Metabolic pathways	1.28	0.69	0.74
Microbial metabolism in diverse environments	1.51	0.63	0.65

Enriched metabolic pathway (downregulated)_negative	Factor	-log10 (FDR)	-log10 (p)
Starch and sucrose metabolism	9.82	7.32	9.06
Galactose metabolism	7.97	7.03	8.46
ABC transporters	2.71	5.72	6.98
Phosphotransferase system (PTS)	4.65	5.05	6.19
Carbohydrate digestion and absorption	15.93	3.10	4.14
Flavone and flavonol biosynthesis	7.12	2.34	3.31
Amino sugar and nucleotide sugar metabolism	1.74	1.66	2.36
Metabolic pathways	1.37	1.65	2.31
Biosynthesis of secondary metabolites	1.81	1.46	1.97
Microbial metabolism in diverse environments	0.18	0.20	0.20

Supplemental Figure 1. Enrichment analysis for the metabolites influenced by selenate

(A) Enrichment analysis metabolites with PC1 > 0.4 from the PCA in positive or negative ion mode.

(B) GO list from enrichment analysis in A.

(C) Enrichment analysis of metabolites with PC1 < -0.4 from the PCA in positive or negative ion mode.

(D) GO list from enrichment analysis in C.

KEGG pathways were used to identify the significantly enriched metabolic pathways using the metabolites with MBROLE3 (https://csbg.cnb.csic.es/mbrole3/index.php). The enrichment factor indicates the relative enrichment of each GO within the analyzed set, with significance assessed by *p*-values and FDR (False Discovery Rate).

Supplemental Figure S2

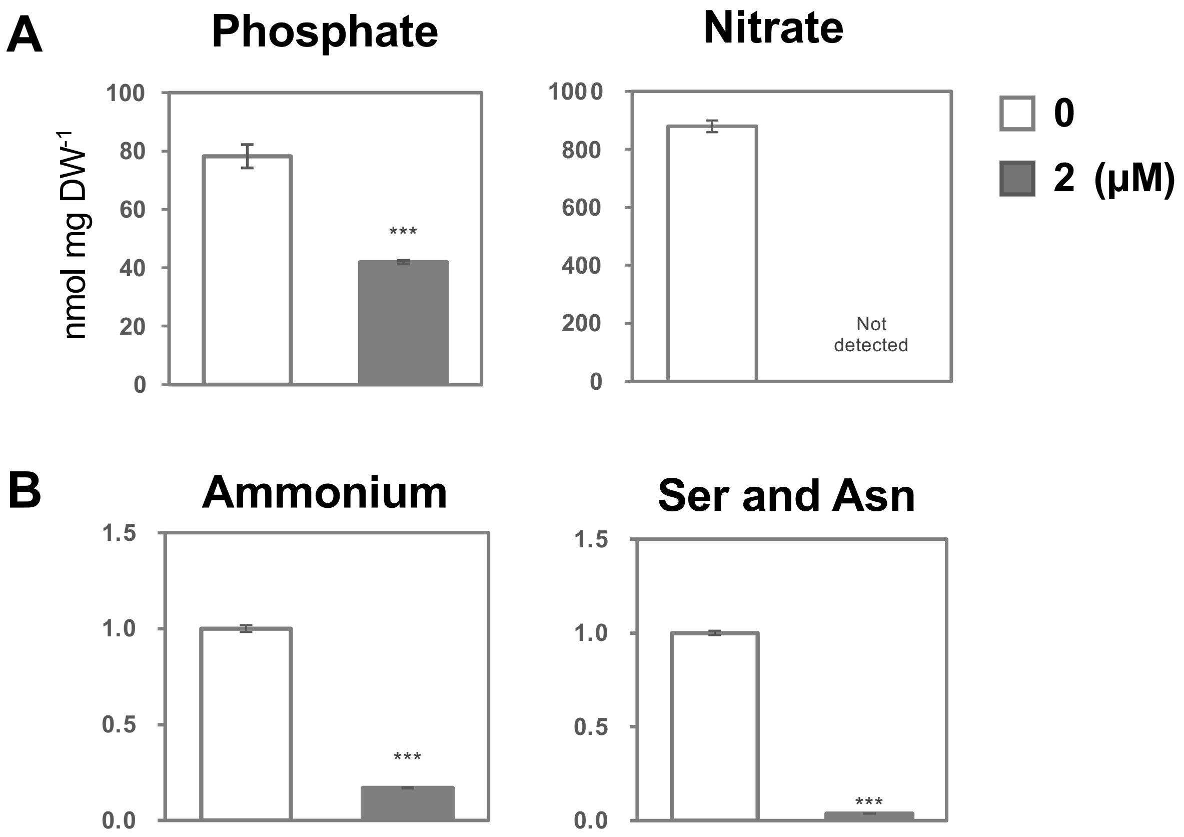

Supplemental Figure S2 Effects of selenate on phosphate, nitrate, ammonium, serine, and asparagine levels in plants
(A) Phosphate and nitrate levels were measured with ion chromatography.
(B) Ammonium, serine, and asparagine relative levels were measured with HPLC.
Bars and error bars represent the mean and standard error (n = 3). Asterisks indicate significant differences between the two conditions as determined by Student's *t*-test (* $0.1 \le p < 0.05$, ** $0.01 \le p < 0.05$, and *** $p < 0.01$).

Supplemental Figure S3

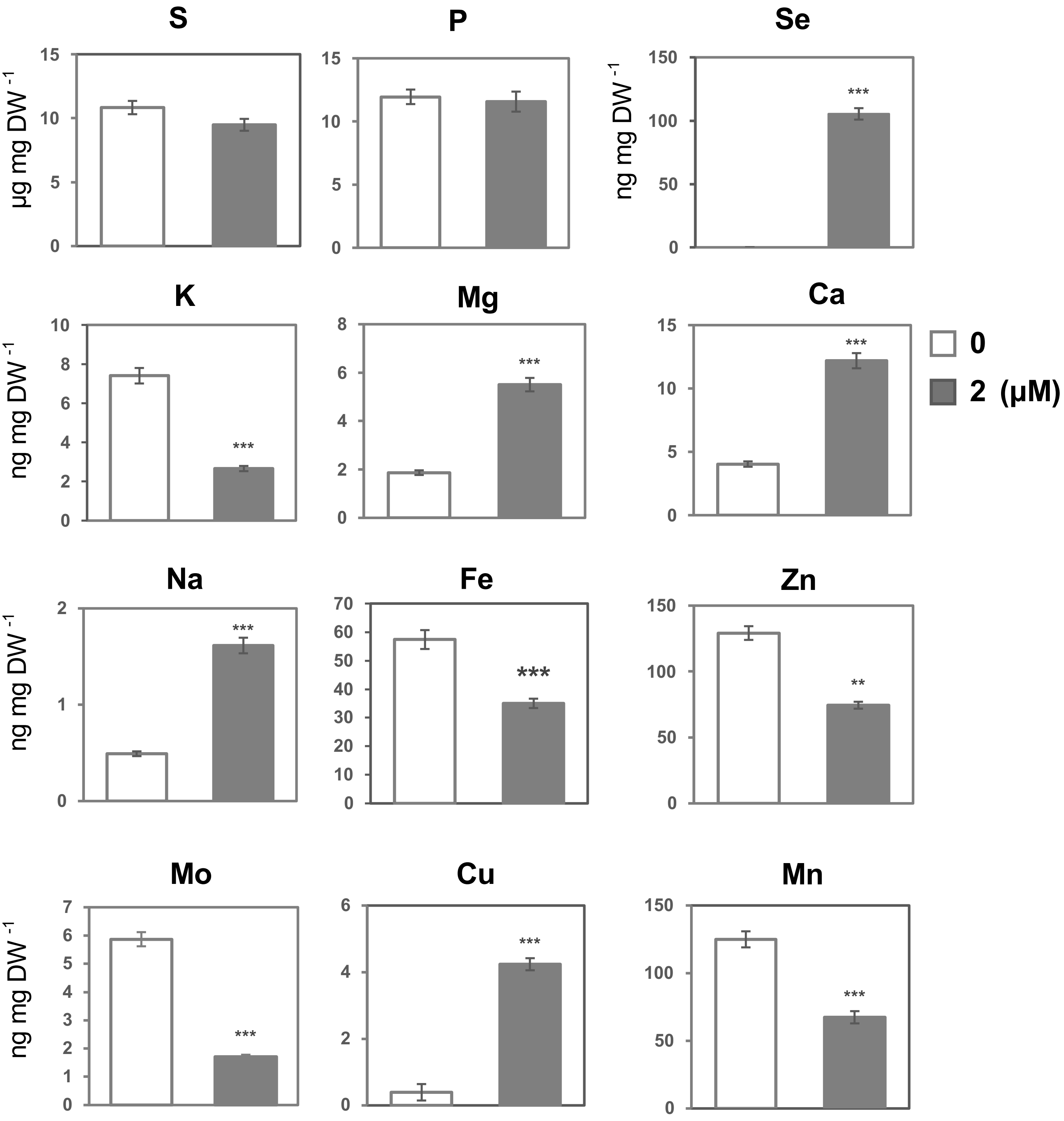

Supplemental Figure S3 Effects of selenate on element levels in plants
Element levels were measured with ICP-OES.
Bars and error bars represent the mean and standard error (n = 3). Asterisks indicate significant differences between the two conditions as determined by Student's *t*-test (* $0.1 \leq p < 0.05$, ** $0.01 \leq p < 0.05$, and *** $p < 0.01$).

Supplemental Figure S4

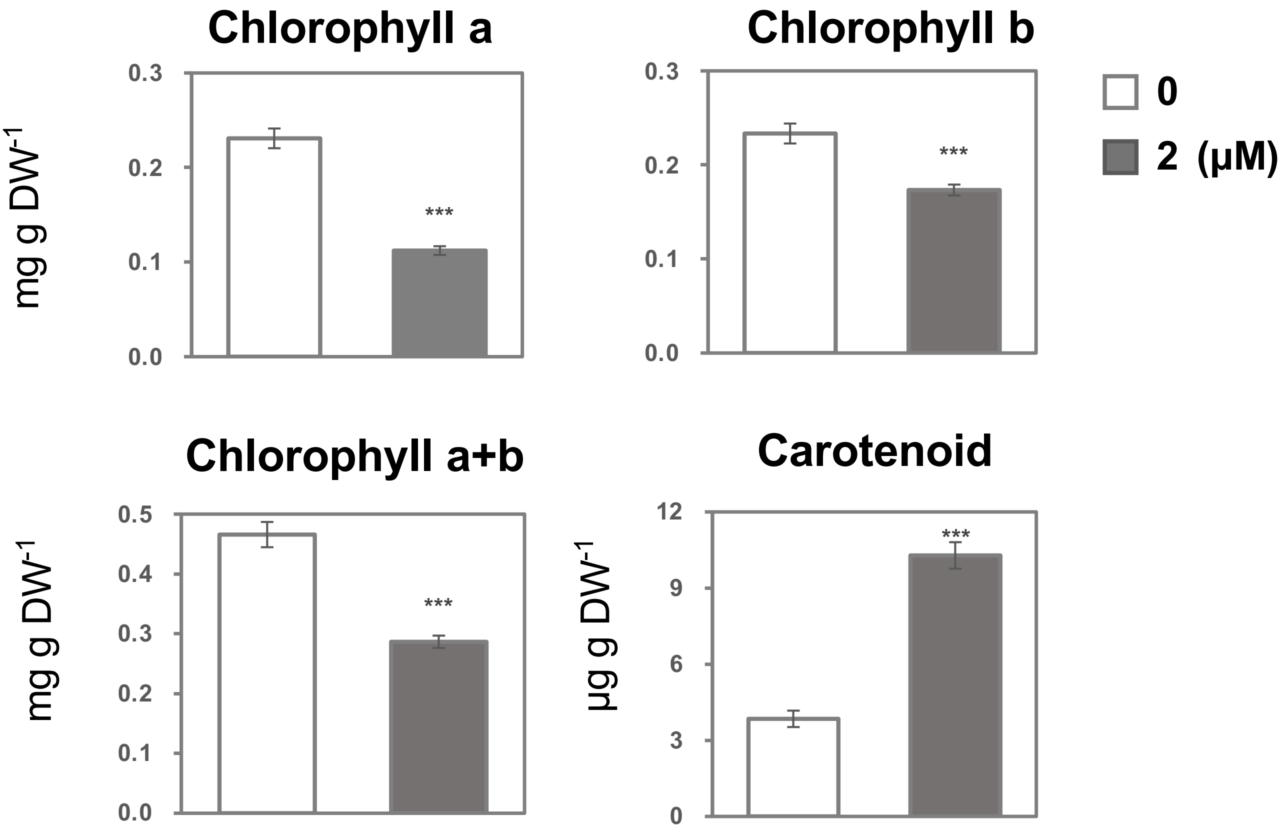

Supplemental Figure S4 Effects of selenate on chlorophyll a, chlorophyll b, and carotenoid levels in plants

Bars and error bars represent the mean and standard error (n = 3). Asterisks indicate significant differences between the two conditions as determined by Student's t-test (* $0.1 \le p < 0.05$, ** $0.01 \le p < 0.05$, and *** $p < 0.01$).
